# Trust-Based Intelligent Routing Protocol with Q-Learning for Mission-Critical Wireless Sensor Networks

**DOI:** 10.3390/s22113975

**Published:** 2022-05-24

**Authors:** DooHo Keum, Young-Bae Ko

**Affiliations:** 1 LIG Nex1 Company Ltd., Seongnam 13488, Korea; dooho.keum@lignex1.com; 2 Department of AI Convergence Network, Ajou University, Suwon 16499, Korea

**Keywords:** mission-critical wireless sensor network, trust-based routing, Q-learning, reinforcement learning, QoS

## Abstract

Mission-critical wireless sensor networks require a trustworthy and punctual routing protocol to ensure the worst-case end-to-end delay and reliability when transmitting mission-critical data collected by various sensors to gateways. In particular, the trustworthiness of mission-critical data must be guaranteed for decision-making and secure communications. However, it is a challenging issue to meet the requirement of both reliability and QoS in sensor networking environments where cyber-attacks may frequently occur and a lot of mission-critical data is generated. This study proposes a trust-based routing protocol that learns the trust elements using Q-learning to detect various attacks and ensure network performance. The proposed mechanism ensures the prompt detection of cyber threats that may occur in a mission-critical wireless sensor network and guarantees the trustworthy transfer of mission-critical sensor data. This paper introduces a distributed transmission technology that prioritizes the trustworthiness of mission-critical data through Q-learning results considering trustworthiness, QoS, and energy factors. It is a technology suitable for mission-critical wireless sensor network operational environments and can reliably operate resource-constrained devices. We implemented and performed a comprehensive evaluation of our scheme using the OPNET simulator. In addition, we measured packet delivery rates, throughput, survivability, and delay considering the characteristics of mission-critical sensor networks. The simulation results show an enhanced performance when compared with other mechanisms.

## 1. Introduction

Mission-critical wireless sensor networks (MC-WSNs) are environments that support mission-critical operations such as emergency response, battlefield surveillance, and large-scale data collection. In most applications, sensors need to support various critical missions, such as fire alarms and battlefield environment detection [1]. To support MC-WSN characteristics, tasks must be executed flexibly with limited bandwidth and energy. Moreover, such networks must have a high time efficiency to handle critical missions in a timely manner. They must also be sufficiently trustworthy to defend against attacks [2]. Thus, mission-critical applications should be able to ensure the worst-case end-to-end delay and reliability when transmitting data to gateways from sensors such as biosensors, temperature sensors, and nuclear sensors.

However, a large amount of data can be generated in an emergency, and it is challenging to guarantee network stability owing to environmental changes. Therefore, it is essential to design a routing protocol that guarantees reliability by considering the characteristics of the sensor and network conditions. When the amount of significant sensor data increases, the network may be exposed to various cyber threats by malicious nodes. When the malicious action of stealing or dropping mission-critical data occurs, it becomes challenging to transmit the sensor data to the gateway, which can significantly affect the mission operation. To solve these problems, studies that consider trust evaluation, end-to-end delay, and energy as metrics are actively being conducted. Recently, research on learning these metric elements to improve reliability has also attracted attention.

This study proposes a trust-based routing protocol that learns the trust elements using Q-learning to detect various attacks and ensure performance. Machine learning methods can be typically classified into supervised learning/unsupervised learning/reinforcement learning. Q-learning, a well-known reinforcement learning (RL) method, can be a useful technique for achieving adaptive trustworthiness in harsh environments [3,4]. It considers the history of significant testing results from previous data reporting intervals. The reason we use Q-learning is that it is appropriate to receive a reward for every hop while transmitting data from the source node to the destination. As a result, the trust reputation for each sensor node is determined using Q-learning, which helps identify malicious nodes [5]. The proposed scheme is divided into local trust evaluation and global trust evaluation with Q-learning to satisfy the mission-critical application requirements and to discover the most trusted path. The proposed technique focuses on how to quickly and securely transmit mission-critical data to its destination by learning as well as calculating trustworthiness and QoS factors.

We propose a distributed transmission technology that prioritizes the reliability of mission-critical data through the Q-learning result considering trustworthiness, QoS, and energy factors. This method can solve the problem of reduced network performance when data is transmitted over a single path. The proposed distributed transmission method ensures reliability by transmitting mission-critical data with the highest priority to the path of MAX-Q value and transmits data of relatively low importance through the suboptimal path that satisfies each requirement. This method can satisfy the requirements of all mission-critical data while ensuring the reliability of the most important data first. In addition, it is possible to stably operate devices with limited resources with technology suitable for mission-critical wireless sensor network operating environments by proposing flexible weights in consideration of energy. We propose a flexible threshold considering the link condition to detect malicious nodes effectively. It is possible to detect malicious nodes according to data occurrence and attack situations effectively.

For performance evaluation, we measure packet delivery rate, throughput, survivability, and delay according to simulation time and malicious node rate. The proposed technology resulted in a higher packet delivery rate and throughput compared to other schemes and confirmed the low end-to-end delay. In addition, the survivability of the node was confirmed to be superior compared to other schemes. As a result, the proposed technology has proven to be an optimal solution for mission-critical wireless network environments.

### 1.1. Motivation

The purpose of almost multipath routing protocols is to use multipath as a substitute route when the network link is broken [6]. However, if large amounts of data are being transmitted, transmission over a single alternative route can be difficult to attain using only bandwidth management and queuing techniques. In addition, advanced attacks such as unplanned on-off are still difficult to detect and prevent the achievement of targeted missions. For these reasons, the requirements for mission-critical data may not be met, which can have the worst consequences for mission operations.

We attempt to ensure trustworthiness and QoS by transmitting the data through all multiple possible routes that satisfy the requirements of each packet. Our mechanism ensures the successful detection of malicious nodes using flexible methods in MC-WSN and guarantees the trustworthy transfer of data. The proposed technology can provide clearer and more accurate trust evaluation through Q-learning in MC-WSN and transmit mission-critical data. As a result, The MC-WSN gateway quickly and correctly gather mission-critical data vital for situational awareness and operations.

### 1.2. Contributions

The major contributions of our mechanism can be summarized as follows:

- Design of an intelligent Q-learning based routing protocol by considering both trustworthiness and QoS (Quality of Service) requirements for MC-WSNs
- Design of a flexible threshold mechanism that contemplates link bandwidth and data usage to increase the probability of malicious node detection.
- Efficient energy management of resource-constrained sensor nodes by placing flexible weights according to the node’s status.
- Proposal of a trust evaluation method and intelligent routing protocol that meets the requirements of mission-critical applications.

The remainder of the paper is as follows. We will review related works in Section 2. In Section 3, we present our proposed mechanism in detail. The performance evaluation is discussed in Section 4. Finally, we conclude our paper and arrange the summary in Section 5.

## 2. Related Work

Trust-based routing is a scheme that discovers and maintains trusted paths by using a measure of trustworthiness derived from trust evaluation. In this section, we review existing trust-based routing methods that can be utilized in MANETs and WSNs. AOTDV [7] is a trust-based multipath routing method that extends AOMDV [8], which maintains multiple paths during the route discovery phase. AOMDV is one of the representative multi-path routing protocols and proposes a method for creating loop-free multi-paths. However, it is vulnerable to attacks by malicious nodes because it focuses on delivering data without considering the trustworthiness factor. AOTDV has been further proposed to solve these problems.

In AOTDV, a source node can create multiple loop-free paths to a destination during the route discovery process. A destination will respond with at most k shortest paths as candidates that satisfy the trust requirements of data packets. Each node has trust evaluation results composed of trust values and a hop count. After the trust path has been created, the source node allocates paths that satisfy the trustworthiness required to generate mission-critical data packets and then generates the data. As an intelligent agent, each node evaluates its neighbors’ behaviors and selects the shortest trusted path to forward packets. However, this scheme cause problem like bottlenecks because it can allocate only a single path for transmitting mission-critical data even though multiple paths are kept for an alternative when the allocated path becomes failed to use. In addition, AOTDV considers trustworthiness and hop count factors as a routing metric, so it is difficult to guarantee the QoS requirements of mission-critical data or the survivability of sensor devices.

TQR [9] proposed the view of trust and QoS metrics evaluation to create a trust-based QoS model. They introduce the definition of trust and QoS parameters estimation into a classic routing to enhance the security of networks. The proposed trust model obtained the degree of trust between nodes from the direct and indirect trust. In addition, due to the NP-completeness of the multi-QoS constraints problem, they only consider the link delay as the QoS constraint requirement for establishing trusted routing. They measured the expected transmission count (ETX), trustworthiness, transmission delay, and propagation delay in calculating the metric. They focus on exploring a feasible way to estimate the available link delay requirement by considering link quality and incorporating a trust-aware scheme into the route discovery procedure to enhance the security of the network. However, the delay cumulated in the queue was not regarded, and only a single path was used for data transmission, making trustworthy communication challenging. TQR considers trustworthiness and QoS factors as routing metrics, but it is difficult to derive accurate values without learning historical data. Moreover, the survivability of the sensor device can be reduced because the energy factor is not taken into account.

CENTERA [10] uses the powerful gateway to collect trust values from individual nodes and evaluate the best paths after isolating “malicious” nodes. Gateway establishes a global view of the network and evaluates three quality metrics: maliciousness, cooperation, and compatibility. The gateway estimates the battery of every node-based and evaluates several metrics for every node. Gateway periodically collects trust values from the individual nodes about the number of packets sent through neighbors. Gateway utilizes a method to disseminate updated routing information to all the network nodes such that each node knows its uplink nodes to forward their packets to the gateway and its next-hop downlink node forwards its own packets through it. However, CENTERA considers only the packet forwarding ratio to perform the trust evaluation of the individual nodes. CENTERA is not suitable for mission-critical wireless sensor networks because it is vulnerable to denial of service or on-off attacks and does not consider the priority of data.

SQEER [11] is devised based on energy and trust modeling for enhancing the security of WSN and also to optimize the energy. The trust modeling utilizes a technique with a security mechanism for providing trust scores. A cluster-based trust routing protocol is designed in which the cluster head has been selected based on trust scores and QoS metrics to perform trust routing. In addition, trust scores, namely direct, indirect and overall trust scores, are evaluated for enhancing the security of communication. The final path algorithm has been allocated based on energy, path trust, and hop count to carry out the secure routing process. However, SQEER is limited in the MC-WSN environment because it transmits data by assigning it to a single path without considering the priority according to the importance of the mission of the tactical data. Moreover, it is not suitable because there are no considerations to satisfy the performance requirements of mission-critical data.

TBSIOP [12] utilizes three distinct WSN attributes to compute a node’s likelihood of being malicious. These attributes are in energy depletion, acknowledgment, and forwarding data packets, and these attributes are used for trust computation. TBSIOP deals with the selection of the best relay candidate among the potential forwarder candidates. The potential forwarder node will be chosen from a set of forwarder nodes based on trust values and probabilities of being selected from the forwarder list. TBSIOP proposes a method of selectively transmitting specific data without classifying mission-critical data. In this case, large-capacity data cannot be processed and the QoS factor of mission-critical data is not satisfied.

ATRP [13] has been proposed that encompasses direct trust, indirect trust, and witness trust, considering multiple factors (resources and security) in its trustworthiness and using pairwise comparison. The proposed mechanism allows further evaluations of additional potential nodes at several hops, which helps to balance the energy consumption and prolong the network lifetime. The objective of the ATRP is to propose an efficient trust-based routing protocol for the selection of relay nodes in distributed WSNs based on multiple trust factors and multilevel trust evaluations using the Q-learning technique. The protocol employs multiple evaluations at multiple layers rather than single-hop evaluations. The consideration of multiple factors in the ATRP balances the load distribution in the network and provides a more accurate selection of the next forwarder. In the proposed approach, both successful and failed transmissions contribute to the calculation of the Q-values. The control mechanism unit in the ATRP is responsible for ensuring that the trust value is valid and reliable. There are three components in the control mechanism unit: number of interactions, decay time factor, and timeliness measurement. However, it is difficult to detect intelligent behaviors such as on-off attacks using this scheme, and it is hard to guarantee the QoS of mission-critical applications by proposing an energy-oriented reward function. In addition, ATRP is a protocol that is not suitable for operation in a mission-critical wireless sensor network environment because it does not sufficiently consider a method to satisfy the requirements of mission-critical data.

Compared with such existing methods, trust-based intelligent routing is novel in terms of three aspects. First, a mission-oriented path allocation algorithm is proposed to meet the requirements of trustworthiness and QoS in MC-WSNs. Second, we suggest a Q-learning-based trust routing technology that detects clever attacks and ensures the reliability of mission-critical applications. Third, we propose a flexible weight for the energy and detection ratio in MC-WSNs. The proposed method can detect and exclude various cyber-attacks through a learning method and a flexible blacklist threshold method. Additionally, it can solve bottleneck problems and ensure the reliability of mission-critical data by using effective distributed multi-path routing.

## 3. Proposed Scheme: MC-TIRP

This section proposes a mission-critical trust-based intelligent routing protocol (MC-TIRP). The proposed scheme consists of several components for trusted route discovery, trust evaluation, and trust update and maintenance. This section also introduces the process for deriving trust values from MC-WSNs as the basis of the discovery and maintenance of a trustworthy and reliable path using Q-learning. As a first step, the proposed algorithm performs the process of discovering multiple paths, excluding malicious nodes based on the trust evaluation value. After finding a trust-based multi-path, Q-learning is performed by considering trustworthiness, QoS, and energy factors to transmit mission-critical data in a distributed manner. In addition, learning results reflect historical records, so it can effectively counteract malicious nodes that perform on-off attacks at specific times. The algorithm operates in a distributed transmission method that sacrifices data of relatively low importance to ensure the reliability of data transmission with high mission importance in consideration of the PBAS (precedence based assured service) concept. Finally, the trust route maintenance and update phase manages multiple routes and processes data. This step ensures distributed data transmission while periodically managing and maintaining the calculated trust value for multipath. It also enables flexible response in case of network problems. We define the notation of the proposed scheme in Table 1.

### 3.1. Trusted Route Discovery Component

In this component, a route discovery method works similarly to the basic AOMDV. However, there are differences in the method processes. Nodes perform direct observations, indirect observations, and witness observations to find a trusted route. Trust discovery works closely with trust evaluation to share the necessary trust and learning values. The source node broadcasts the RREQ (Route Request) to find trusted paths to the gateway node. Intermediate nodes receiving the request message reply to the source node with trust information. The nodes check the blacklist, except the malicious node. The intermediate node considers the trust value and multiple loop-free route discovery processes and broadcasts the RREQ. When the destination receives the RREQ message, it responds with the RREP (Route Reply) and reward. In a wireless environment, more routes may exist according to various situations, and this can be set according to the operator’s judgment. In the paper, we set up to maintain a maximum of five valid trust paths. The reason is that we assumed five types of mission-critical data to be distributed over a trusted path. The source node can transmit data over trusted paths formed on the basis of Q-learning. Mission-critical data can be transmitted over a path with the maximum Q-value. Data of relatively low mission importance can be transmitted randomly over trustworthy paths. We presumed that data packets A and B had high priority, and packets C, D, and E had a relatively low priority; however, the definition could be obtained according to the preferences and intentions of the operator.

### 3.2. Trust Evaluation Component

In this paper, trust evaluation is classified into local trust evaluation and global trust evaluation. First, local trust evaluation derives local trust based on the trustworthiness, QoS, and energy value evaluated by each node and learns by receiving it as a reward. This method detects and excludes malicious nodes through flexible thresholds in the process of deriving local trusts and transfers mission-critical data to a trusted alternative path. It also proposes a flexible weighting method to ensure reliability while managing the energy of resource-constrained devices. Global trust evaluation is calculated based on the local trust value of the nodes included in the multi-path, and it learns by deriving a global trust for the entire path and receiving it as a reward. As a result, the optimal path can be learned and the data with the highest mission criticality can be transferred to a safe path.

#### 3.2.1. Local Trust Evaluation

Trust evaluation is derived by learning based on the results of the discovery component. The sensor nodes monitor the behavior of neighboring nodes and apply a formula that considers the packet forwarding ratio (*PFR*), expected transmission time (*ETT*), and energy.

(1) $$PFR_{i,\;j}=\frac{F_{i,\;j\;}\left(t\right)}{S_{i,\;j\;}\left(t\right)}$$

*PFR* is derived by observing the packet-forwarding behavior of neighboring nodes using the promiscuous mode, as shown in Equation (1). $S_{i,\;j\;}\left(t\right)$ is the number of packets sent by node *i* to node *j*, and $F_{i,\;j\;}\left(t\right)$ is the number forwarded by node *j*. This method can detect nodes maliciously dropping packets through *PFR* calculation and simply obtain the reliability of the link. However, a simple method of calculating *PFR* is difficult to detect advanced attacks such as on-off that are performed according to a specific time. We utilize a flexible blacklist threshold and Q-learning to address this. A blacklist threshold value (γ) is defined and is used to identify malicious nodes (0≤γ≤0.75). The blacklist threshold considers the mission-critical tactical network of nodes and is measured as shown in Equation (2).

(2) $$Blacklist\;threshold\left(\gamma \right)=\frac{Link\;bandwidth}{Current\;bandwidth}$$

*Link bandwidth* is the maximum rate of data transfer across a given link. The current bandwidth is an estimate of the *Current bandwidth* of the network interface in bits per second (bps). The reason for the above calculation is to determine whether an attack by a malicious node is occurring or the communication status is bad. Low *Current bandwidth* usage increases the blacklist threshold to detect malicious nodes with low drop attacks. High *Current bandwidth* usage makes it difficult to determine whether an attack from a malicious node or a bottleneck is causing network performance degradation. The blacklist threshold is reduced to help in the discreet resolution of malicious node exclusions [14].

Trust evaluation is classified into types: direct, indirect, and witness trust [13]. Direct trust is derived by evaluators by observing the behavior of their direct neighbor nodes. Indirect trust is the recommended value calculated for the target node obtained from the evaluator’s indirect node. When it is difficult to perform an accurate trust evaluation using only direct trust, it is possible to perform a more accurate trust evaluation from calculations based on recommendations received from indirect nodes. The indirect trust value for calculating the total trust value is forwarded from the direct node to the source node. A witness trust is a value by which a proven direct node evaluates an indirect node and recommends the value to the source node. The difference between indirect and witness trusts is that the indirect trust is calculated and recommended by indirect nodes, whereas the witness trust is done by certified direct nodes. We consider not only direct trust but also indirect witness trust for a more accurate and effective trust evaluation. The QoS factor of links is measured using *ETT*, as shown in Equation (3).

(3) $$ETT=ETX\times \frac{X}{B}$$

*ETT* improves *ETX* (Expected Transmission Count) by considering the differences in link transmission rates. *ETT* is an appropriate QoS factor to satisfy the delay requirements of mission-critical data. *X* denotes the size of the packet (for example, 1024 bytes), and *B* denotes the bandwidth (raw data rate) of the link [15]. *ETT* does not include the back-off time spent waiting for the wireless channel [16].

In MC-WSN, if the devices cannot operate due to an energy discharge, it can have a fatal impact on operations. Therefore, it is essential to calculate the reliability evaluation considering energy. Resource-constrained devices that operate in a WSN environment require recharging or exchange when their energy is depleted. The proposed technique calculates the energy required for a resource-constrained device, as shown in Equation (4) below, where $ERes_{i\;}$ is the residual energy of node *i*, and $EInit_{i\;}$ is the initial energy of node *i*.

(4) $$Energy=\frac{ERes_{i\;}}{EInit_{i\;}}$$

Using these derived factors, the node with insufficient residual energy calculates the energy-based QoS and trust value (*EQTV*), as shown in Equation (5). The *EQTV* equation calculates which metrics to prioritize: trustworthiness, QoS, and energy by weighting them based on the node’s energy state.

(5) $$EQTV=\left(\omega_{1}\times Energy\right)+\left((\omega_{2}\times \left(PFR\times \left(1-ETT\right)\right)\right)$$

(6) $$\omega_{1}=\left(1-Energy\right),\;\omega_{2}=\left(1-\omega_{1}\right)$$

In this study, we propose a flexible weight module to distribute weights adaptively according to the status of a device. For this purpose, a flexible weight module that efficiently manages energy is essential. In a sensor network composed of trusted nodes, this technique applies a flexible weight based on the remaining energy. $\omega_{1}$ is determined based on the residual energy, and $\omega_{2}$ is calculated using Equation (6) based on $\omega_{1}$. A node with sufficient energy assigns a higher weight to reliability and QoS, and a node with insufficient energy assigns a higher weight to energy. Therefore, it is possible to enhance survivability using an energy-based flexible weighting method.

The derived trust value is calculated as the local trust level by applying the formula below. Local trust is the trust-based value of the k neighbor nodes of node *j* and is calculated as shown in Equation (7). Local trust is calculated as the average of *EQTV* values calculated by neighboring nodes, and it is possible to evaluate local trust more accurately. *n* denotes the total number of *k* neighbor nodes and is derived by averaging the trust values evaluated by *n* nodes.

(7) $$LocalTrust=\frac{1}{n}\overset{n}{\underset{k=1}{\sum }}EQTV_{k,\;j}\left(t\right).$$

#### 3.2.2. Global Trust Evaluation

Global trust evaluation is a method of receiving a reward from a destination node when the mission-critical data requirements are satisfied. Each mission-critical application has different requirements that must be met [14,17,18]. Therefore, although local trust evaluation between nodes is vital, a reward system is activated when the requirements along the entire path are satisfied. The global trust provides the average value of local trust, as shown in Equation (8). The global trust is calculated as the average of the local trust values of the nodes included in each path, and as a result, the trust value of the entire path can be obtained.

(8) $$GlobalTrust=\frac{1}{n}\overset{n}{\underset{k=1}{\sum }}LocalTrust_{p}\left(t\right).$$

In the case of end-to-end delay, global trust is compensated when the communication requirements of IoT applications are less than 100 ms [16] and the *PTV* (Path Trust Value) is higher than 0.65 [7] (0 ≤ PTV ≤ 1). We assign the minimum *PFR* of the nodes in the trusted route to the *PTV*, as shown in Equation (9) [19,20]. *PTV* values can verify that the measured path trust values meet the trust requirements of mission-critical data.

(9) PTV=minPFR

In this global trust evaluation, the reward is calculated when the packet sent from the sensor node to the destination satisfies the mission-critical data requirements. This ensures the reliability of data that meets the requirements of mission-critical applications.

#### 3.2.3. Q-Learning Based Trust Evaluation

Q-learning is a form of model-free RL. RL is formulated using the Markov decision process (MDP) and is defined as a quintuple (S,A,E,T,R), where S is the set of states of the system, A is the set of actions performed by the agent that affects the system, E is the set of external events that the agent has no control over, and T is the transition that connects each state, action, and event. R is the reward that the agent receives for taking action [3,21]. A trust value is updated using the Q-learning technique, in which the agent learns an action-value function, labeled Q (state, action), that describes the value of performing the action in state *s*. Q-learning is implemented using Equation (10) [22,23]. Initially, all Q tables are set to 0, and to update the Q value, each node evaluates the trust of neighboring nodes.

(10) $$Q\left(s,a\right)=\left(1-\alpha \right)\times Q\left(s,a\right)+\left(\alpha \;\times \;\left[Reward+\mu \times MAX\;Q\left(s^{\prime },\;a^{\prime }\right)\right]\right).$$

We define the state s as the trust status of each node and update it with rewards according to the calculated local and global trust values. In addition, to reflect the trust state of nodes and networks, the alpha ratio α is set to 0.5, and the decay factor μ is set to 0.9. In particular, if a random action is frequently selected, the network performance may be degraded, so epsilon is set to lower the random probability. The Q value is calculated based on the trust value for each neighbor. The action a may be selected randomly according to the epsilon value or according to the maximum Q value. The reward is received according to the action result, and a new Q value is updated based on the Q-learning algorithm. In the proposed approach, local trust can be calculated through trust information periodically received from neighboring nodes. When the data packet arrives at the gateway, global trust is applied as a reward.

**Figure 1:**
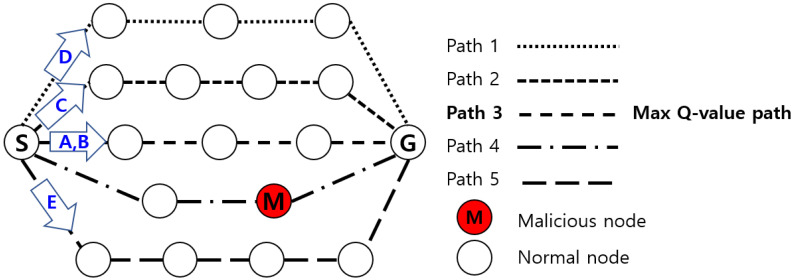
Example of data transmission based on mission-critical data priority.

Figure 1 shows an example of data transmission based on the priority of mission-critical data. The source node excludes the path where the malicious node M is detected via trust evaluation and derives Q-values for each path based on local and global trust. The source node then checks the Q-values of all paths and executes an algorithm to transmit mission-critical data. Notice that mission-critical data A and B are transmitted over the route with the max Q-value (Path 3). C, D, and E data of relatively low importance are randomly transmitted among paths that satisfy each network requirement. As a result, mission-critical data is transmitted through a path that guarantees reliability and QoS while satisfying the requirements of each data.

### 3.3. Trust Route Update and Maintenance Component

**Figure 2:**
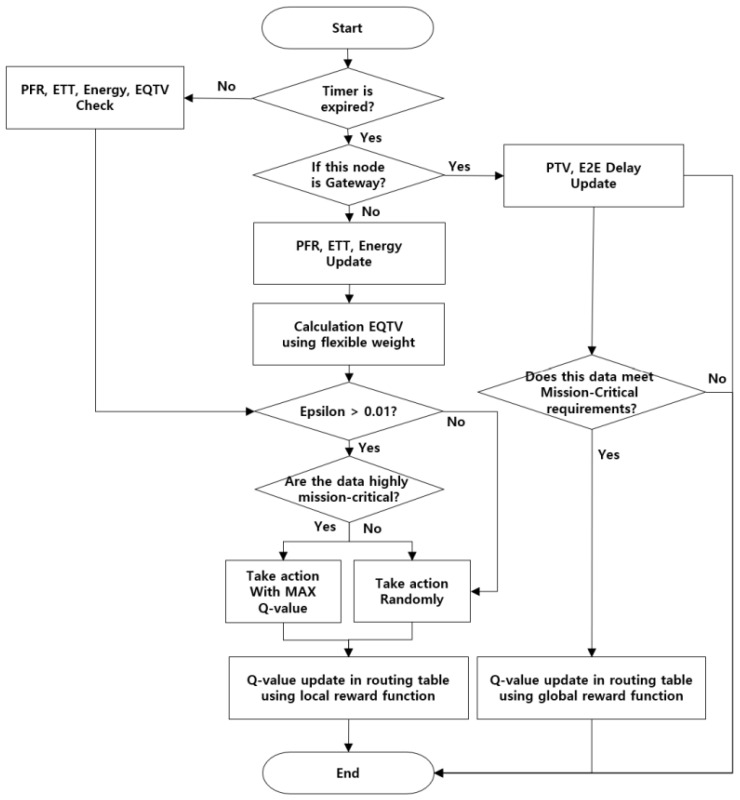
The overall process for MC-TIRP Scheme.

Path update and maintenance is the process of deciding how to update or maintain routes if network status changes (e.g., data usage, link status, or the event of link disconnection). If the maintenance process identifies nodes that cannot function, it transmits an error message to the source node. If the source and intermediate nodes have active multipaths, they are updated to alternate paths, and transmission takes place. If there is no trust path, the source node can execute a route discovery routine to discover a new trust route. The path maintenance process validates the trusted path at certain time intervals. When a trust route cache entry exceeds its validity time, a new trust route retrieval process is started. Figure 2 shows a comprehensive flowchart of the trust-based intelligent routing protocol scheme. Each sensor node calculates *EQTV* using flexible weights. To learn a reliable path, the trust values of nodes and paths are periodically checked and updated using a hello message with *EQTV*. After that, if the Epsilon value is above a certain level, we check the importance of the mission data. The reason for choosing the Epsilon criterion is that selecting random actions too often can degrade network performance. If the data to be transmitted has high mission criticality, the max-Q value path is selected; otherwise, a random path is selected. Finally, sensor nodes update the local trust value in the routing table and learn as a reward.

The gateway node updates the PTV and end-to-end (E2E) delay of the paths and receives a global reward when the mission-critical requirements are satisfied. Finally, the gateway node updates the global trust value in the routing table and learns as a reward. As a result, the Q table of the path that satisfies the requirements is learned to enable efficient path management.

## 4. Performance Evaluation

We introduce the simulation environment settings, as described in Table 2. The proposed protocol (MC-TIRP) was implemented using the OPNET and compared with the ATRP, TQR, and AOTDV. The simulation was deployed with 100 nodes in a partial mesh topology that was in a 1000 m × 1000 m area. A fixed gateway node and 99 sensor nodes were used. We set the percentage of malicious nodes between 0 and 40% and performed on-off attacks (including DoS and gray hole attacks). An attacker can launch all the behaviors continuously, with 70% good behaviors and 30% bad behaviors [24,25]. Malicious nodes performing grey hole attacks drop packets at a rate of 30%. A malicious node performing a DoS attack could send a large number of packets to the target node. DoS attacks generate various types of traffic, and the network situation changes dynamically.

We used a constant bit rate (CBR) data traffic model by considering the type, size, and sensor data transmissions in MC-WSNs [26,27,28]. In the experimental environment, a larger amount of data than the link bandwidth capacity was transmitted to mimic the MC-WSN condition. The PHY was set to 2 Mbps to imitate the resource-constrained communication in the tactical network [29,30]. For performance evaluation, the end-to-end delay, throughput, and packet delivery ratio (PDR) were analyzed. Delay refers to the E2E delay from the time the sending node sent the packet to the time the destination node received the packet. Throughput was measured by taking into the number of packets transmitted within a given period. PDR was measured by taking into account the number of packets transmitted and received, and the energy was calculated by determining the initial energy and decreasing it according to the rate of packet delivery.

**Figure 3:**
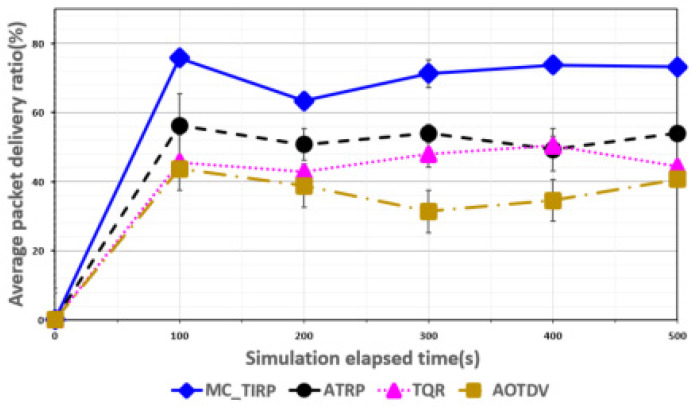
Average PDR over elapsed time.

**Figure 4:**
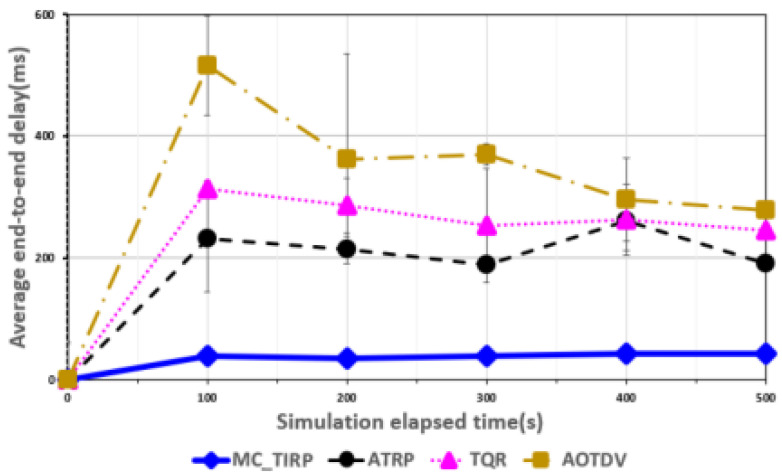
Average E2E delay over elapsed time.

**Figure 5:**
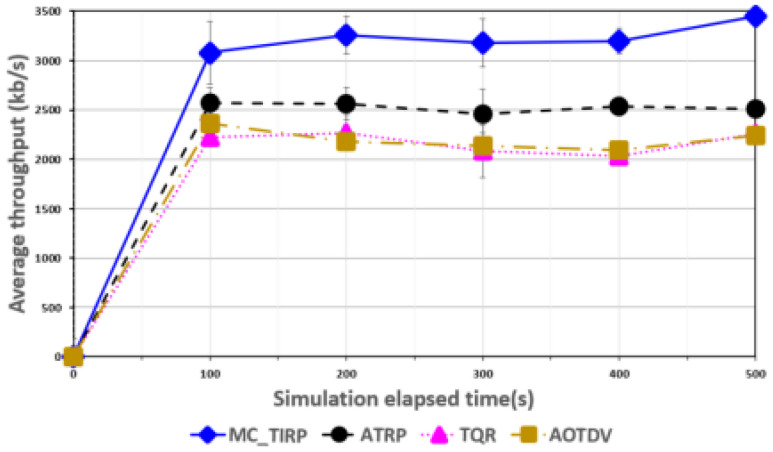
Average throughput over elapsed time.

**Figure 6:**
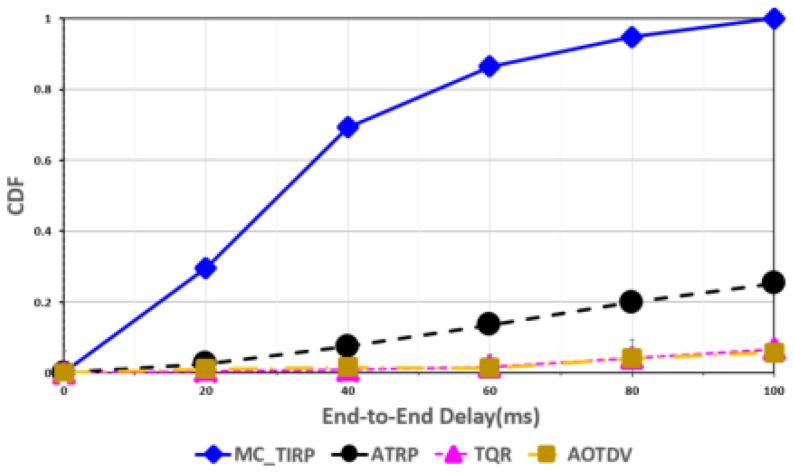
Normalized CDF of end-to-end delay for received mission-critical data A.

**Figure 7:**
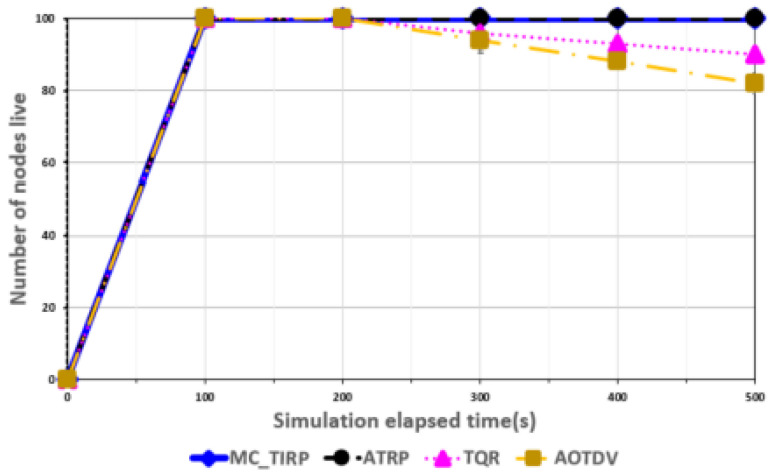
Number of live nodes over elapsed time.

Figure 3, Figure 4, Figure 5, Figure 6 and Figure 7 shows the network performance evaluation when the proportion of malicious nodes is 30%.

Figure 3 shows the average PDR over elapsed time. AOTDV and TQR use trust metrics to select the trust path; however, intelligent DoS attacks that generate various types of traffic are difficult to detect. Damage from on-off attacks was accumulated due to the percentage of malicious nodes (30%). We also set up a situation where a lot of sensor data exceeds the link bandwidth. Therefore, the overall performance of the packet forwarding rate is degraded. In the case of AOTDV and TQR, data transmission over a single path became the bottleneck, resulting in an average PDR of less than 50%. As it is difficult to detect on-off attacks using ATRP, damage accumulates, but the reward function considers only energy; thus, it showed a lower PDR result than the proposed technique. The proposed MC-TIRP scheme allocates trusted multipaths using Q-learning-based routing metrics that consider reliability, QoS, and energy. As a result, MC-TIRP showed a PDR of about 70%, showing the highest level among all mechanisms. Our scheme guarantees the load balancing effect by transmitting high-importance data through the max Q-value path and transmitting relatively low data distributed through other paths.

Figure 4 shows the E2E delay over elapsed time. It is difficult to detect a node performing intelligent attacks using ATRP, but it transmits data distributed through Q-learning considering energy factors; hence, it shows a lower delay than TQR and AOTDV, although the malicious node ratio is 30%. However, The end-to-end latency was around 200 ms as the packet processing time increased at the intermediate node due to the intelligent attack, making it difficult to process mission-critical data effectively. AOTDV and TQR are also ineffective at detecting intelligent attacks; therefore, they are vulnerable to DoS attacks that generate various types of traffic. TQR uses a metric that considers both reliability and QoS factors, resulting in a lower E2E delay than AOTDV. However, it is difficult to guarantee the reliability of mission-critical data because of an E2E delay of about 300 ms. AOTDV shows the highest average E2E delay compared to other mechanisms because it uses only reliability metrics, not considering QoS factors while transmitting data over a single path. MC-TIRP showed the lowest E2E delay compared with the other mechanisms because it was learned considering reliability and QoS factors and distributed transmission according to the priority of mission-critical data.

Figure 5 shows the throughput over elapsed time. Throughput was measured by estimating data transfers in terms of overhead, transmission rate, and packet size over a given time period. MC-TIRP quickly found a reliable path considering trust, QoS, and energy and showed a throughput of about 3200 kb/s. In MC-WSN, data must be transmitted accurately and quickly, and the efficiency of the method was confirmed through throughput. AOTDV and TQR showed similar results of around 2200 kb/s. ATRP is ineffective at detecting on-off attacks, but because it selects a path based on Q-learning, it shows higher throughput results than TQR and AOTDV.

Figure 6 shows the normalized cumulative distribution function (CDF) of the end-to-end delay for mission-critical data A. CDF is calculated as the ratio of the number of received packets. Mission-critical data must satisfy performance requirements and guarantee QoS even in worst-case situations. The results show that MC-TIRP collects and processes large quantities of data faster than other techniques, despite the fact that 30% of the nodes are malicious. This is due to the learning of the trust and QoS elements that effectively excluded the malicious nodes so that the mission-critical data A was transmitted on the trusted paths. In the case of ATRP, it found the trusted path by learning the trust factor, but the CDF of end-to-end delay results was less than those of the proposed method because it rewarded only the energy factor and the priority of mission-critical data was not considered. However, mission-critical data were distributed and transmitted through learning considering the energy factor, resulting in higher CDF results than those of TQR and AOTDV. TQR and AOTDV show significantly lower CDFs according to the end-to-end delay compared to the proposed technique. This is because the data were transmitted using one path, and the DoS attack continuously caused a bottleneck.

Figure 7 shows the results for the number of live nodes over time. To determine the number of live nodes, we set the initial energy constant values in a simulation environment without malicious nodes. To measure the residual energy, the rate was decreased every time a packet was transmitted. MC-TIRP and ATRP reflect efficient energy management because trust learning is performed by considering residual energy as a trust metric. Therefore, according to the elapsed time, all nodes were live to check the possible communication results. As TQR changed the path more frequently while considering reliability and QoS factors, more nodes survived than AOTDV. AOTDV shows the result of fewer nodes surviving because the path did not change significantly when using the reliability metric. It was found that approximately 20% of the nodes did not operate because there was no residual energy. If there are nodes that fail to operate in an MC-WSN, they will not be able to support mission operations, which can have devastating consequences.

**Figure 8:**
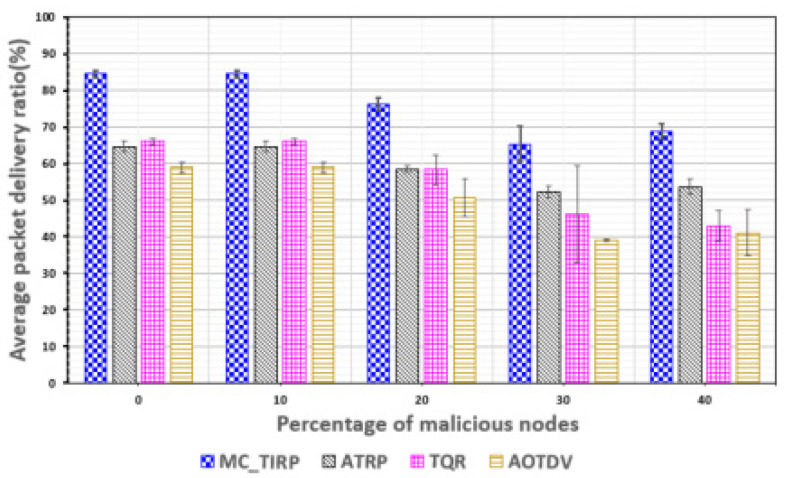
PDR plotted against the proportion of malicious nodes.

Figure 8 shows the PDR versus the percentage of malicious nodes. The result of the percentage of malicious nodes can measure the performance of how quickly they react when attacked. In the MC-WSN environment, the transmission rate of mission-critical data is a very important factor, so accurate performance analysis is required. We measured the PDR by increasing the proportion of malicious nodes from 0 to 40%. The average PDR was calculated by taking into account MC-WSNs where there was a delay due to a bottleneck because of the large amount of mission-critical data usage compared to the link bandwidth. As the proportion of malicious nodes increases, they receive more attacks and increase processing time, which decreases PDR. AOTDV and TQR showed a decrease in the PDR as the percentage of malicious nodes increased. This is because both schemes suggest a method that assigns a single path. MC-TIRP has demonstrated its ability to transmit data more than other methods by using an algorithm that distributes and transmits data after learning a trust metric. ATRP has difficulty in detecting on-off attacks, but because it selects paths based on Q-learning, it shows higher PDR results than TQR and AOTDV.

**Figure 9:**
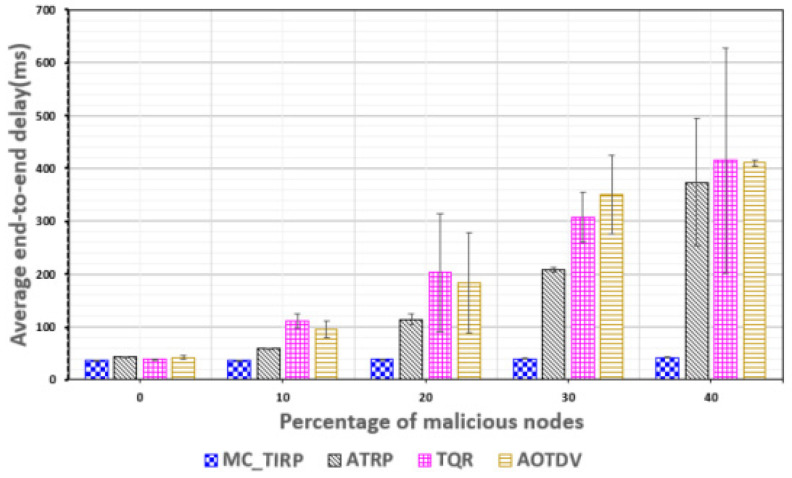
Delay plotted against the proportion of malicious nodes.

Figure 9 shows the E2E delay based on the proportion of malicious nodes. Considering MC-WSN, even without a malicious node, there can be a 40 ms delay because mission-critical data usage is set above the link bandwidth. As a result of the proposed MC-TIRP, the latency did not exceed 50 ms even when the proportion of malicious nodes increased to 40%. The proposed method was verified because the observed delay is lower than the 100 ms delay required in MC-WSN. Among the other schemes, ATRP did not meet the required delay as the proportion of malicious nodes increased. It is difficult to guarantee the QoS factor because ATRP considers only energy for the reward function. However, the mission-critical data were distributed and transmitted through learning considering energy factors, which resulted in a lower average E2E delay than that of AOTDV and TQR. In the case of TQR and AOTDV, which used a method that measured trustworthiness and QoS, a delay occurred because a single route was used for mission-critical data transmission.

**Figure 10:**
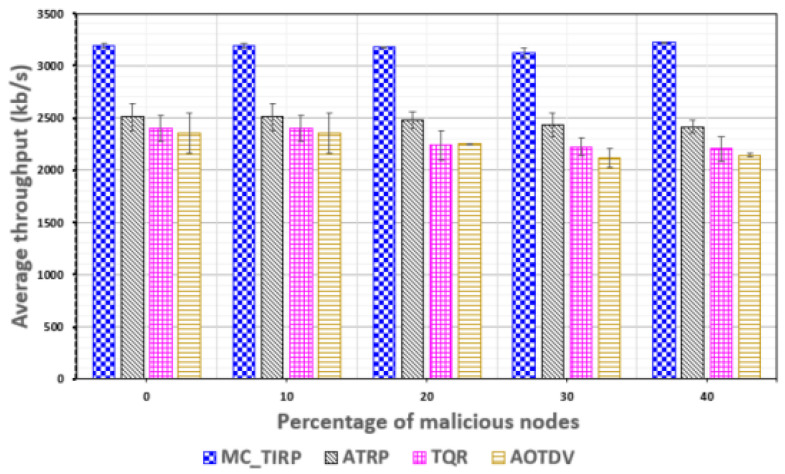
Throughput plotted against the proportion of malicious nodes.

Figure 10 shows the throughput based on the proportion of malicious nodes. Throughput can show similar standard deviation results depending on the situation, even with an increase in malicious nodes. The reason is that processing more large amounts of data at any given time can increase overall average throughput. AOTDV and TQR indicated a throughput of about 2100–2400 kb/s, which decreased when the proportion of malicious nodes increased. It is difficult to maintain throughput in MC-WSNs, which require ensured trustworthiness and transmission importance of data. ATRP uses Q-learning to adaptively select routes, resulting in a throughput of about 2500 kb/s. The proposed MC-TIRP showed a throughput of about 3300 kb/s even when the malicious node ratio increased to 40%, confirming that it can reliably process mission-critical data.

## 5. Conclusions

This study proposes an MC-TIRP that detects malicious nodes in MC-WSNs and transmits mission-critical data through paths that guarantee trustworthiness and reliability. MC-WSN must ensure the transmission of mission-critical data. Therefore, reliability attributes as well as methods to ensure sufficient energy, trustworthiness, and reliability are essential. The technique proposed in this paper supports quick and safe data transmission while fulfilling these requirements. The proposed technique for the survivability of sensor devices focuses on maximizing performance and minimizing calculations for trustworthiness, energy, and delay compared to other technologies. It was found that MC-TIRP demonstrates superior performance in terms of E2E delay, PDR, throughput, and energy when compared to competing mechanisms. In the future, research should be conducted to ensure the trustworthiness of mission-critical data through improved learning and trust evaluation techniques.

## Figures and Tables

**Table 1:** Notation related to the proposed scheme.

Notation	Description
*B*	The bandwidth (raw data rate) of the link
$EInit_{i\;}$	The initial energy of node *i*
*EQTV*	Energy-based QoS and Trust Value
$ERes_{i\;}$	The residual energy of node *i*
*ETT*	Expected Transmission Time
*ETX*	Expected Transmission Count
$F_{i,\;j\;}$	The number forwarded by node *j*
MC-TIRP	Mission-Critical Trust based Intelligent Routing Protocol
*PFR*	Packet Forwarding Ratio
*PTV*	Path Trust Value
$S_{i,\;j\;}$	The number of packets sent by node *i* to node *j*
ω	Weight factor
*X*	The size of the packet
α	Learning rate
γ	Blacklist threshold
μ	Decay factor

**Table 2:** Simulation environment settings.

Parameters	Values
Simulator	OPNET 18.0
Simulation time(s)	500
Routing	MC-TIRP, ATRP, TQR, AOTDV
Number of nodes	100
Percentage of malicious nodes	0–40%
Attack model	Gray hole attack, On-Off attack Denial of Service attack
Traffic Type (Avg. Packet Size)	VoIP G. 723.1 (24 bytes)
	Fire alarm, Chat (100 bytes)
	Health, Temperature, Humidity Sensors (120 bytes)
	Security, Smart Meter (200 bytes)
	Bulk Data, CCTV Camera (2000 bytes)
MAC	CSMA/CA
PHY	802.11b
α	0.5
μ	0.9

## Data Availability

Not applicable.
